# The regulatory landscapes of developmental genes

**DOI:** 10.1242/dev.171736

**Published:** 2020-02-03

**Authors:** Christopher Chase Bolt, Denis Duboule

**Affiliations:** 1 Swiss Institute for Cancer Research (ISREC), School of Life Sciences, Federal Institute of Technology, Lausanne, 1015 Lausanne, Switzerland; 2 Department of Genetics and Evolution, University of Geneva, 1211 Geneva 4, Switzerland; 3 Collège de France, 75005 Paris, France

**Keywords:** Regulatory landscape, Gene regulation, Topologically associating domains, Enhancer-promoter interactions, Pleiotropy, Neo-functionalization

## Abstract

Regulatory landscapes have been defined in vertebrates as large DNA segments containing diverse enhancer sequences that produce coherent gene transcription. These genomic platforms integrate multiple cellular signals and hence can trigger pleiotropic expression of developmental genes. Identifying and evaluating how these chromatin regions operate may be difficult as the underlying regulatory mechanisms can be as unique as the genes they control. In this brief article and accompanying poster, we discuss some of the ways in which regulatory landscapes operate, illustrating these mechanisms using genes important for vertebrate development as examples. We also highlight some of the techniques available to researchers for analysing regulatory landscapes.

## Abstract (precis)

**Summary:** This Development at a Glance article gives an overview of the complexity of regulatory landscapes, highlighting different modes of action for controlling gene expression and the techniques that can be used to study them.

## Introduction

Animal development provides an excellent system in which to study the mechanisms of gene transcription. The dynamic nature of tissue and organ formation requires integration of multiple spatial, temporal and quantitative inputs to progressively define and adapt the properties of cells to their final locations and functional status. A key point during signal integration is at the level of transcriptional regulation, where many fate decisions occur. In the regulatory landscapes of developmental transcription factors, the integration of these events is enacted through the collection of regulatory elements, some of which can act in diverse developmental processes or pathways to produce pleiotropic expression.

**Figure:**
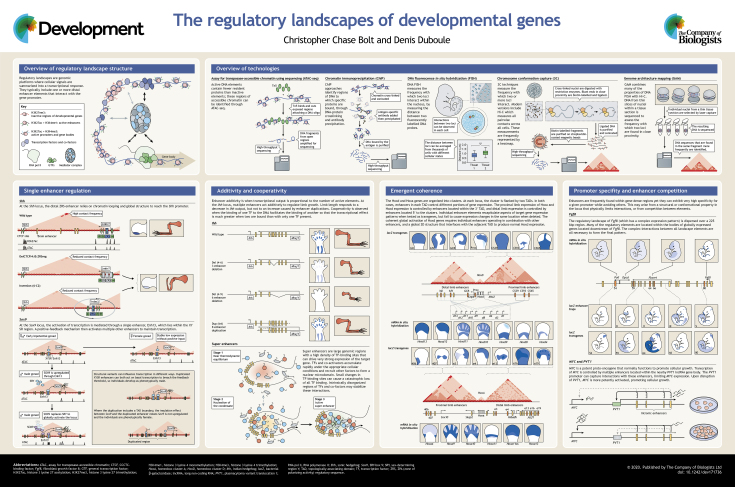

Genes coding for transcription factors (TFs) or developmentally relevant signalling molecules are frequently embedded within large domains of regulation containing multiple control sequences (e.g. Spitz and Furlong, 2012). This can make assigning regulatory elements, such as enhancer sequences, to a particular target gene difficult. Historically, such assignment has been based on controversial assumptions related primarily to the genomic distance between the enhancer and the predicted target gene (Deplancke et al., 2016). In the absence of genetic approaches, this type of analysis can be problematic as the linear size of regulatory landscapes can vary greatly. Recent technological and conceptual developments have made the detection of enhancer sequences and their association with particular genes easier, for example by assaying for specific epigenetic marks or chromatin accessibility (Klemm et al., 2019), and by looking at DNA-DNA interaction profiles. This latter set of techniques has revealed the existence of topologically associating domains (TADs) (Dixon et al., 2012; Nora et al., 2012), which are chromatin structures defined by their increased probability of internal physical interactions. TADs seem to confine the interaction between gene promoters and their flanking enhancers, although this causal relationship remains a controversial issue (as discussed by Mir et al., 2019).

The presence of constitutive chromatin interactions that usually underlie the 3D structures of TADs might permit *de novo* enhancer evolution by providing a ‘chromatin niche’ enriched with appropriate protein content and concentrations to produce the necessary molecular and/or physical micro-environment (Shrinivas et al., 2019). These constitutive niches may have assisted the formation of new productive protein-DNA interactions leading to the evolution of enhancers and the associated neo-functionalization of target genes. In this view, regulatory landscapes and their 3D topologies could also be seen as playgrounds to evolve pleiotropy (Darbellay and Duboule, 2016).

In this article and the accompanying poster, we first introduce the concept of a regulatory landscape, before examining a few examples taken from vertebrate model systems that demonstrate their importance, complexity and variety. We also discuss a few of the possible mechanisms underlying their modes of action. The goal is to illustrate some of the ways that regulatory landscapes operate and how they can be evaluated, not to provide an exhaustive discussion.

## Regulatory landscapes

In our current textbook view of vertebrate transcriptional regulation there are several necessary components: one or more regulatory DNA sequences bound by TFs and co-factors, a gene with its associated promoter, the RNA polymerase machinery, and a factor or factors that bridge and stabilize the aggregate structure and that modulate RNA polymerase release from the promoter (see Long et al., 2016 for a detailed review). The assembly of these components is initiated by heritable and stable properties of the DNA sequence itself, generally referred to as regulatory elements. These are defined as *cis*-acting, generally non-coding DNA elements approximately 1 kb in size that can be found at various distances from their target promoters, and possibly – although not always – act through a proximity-dependent effect that modulates the probability of transcriptional initiation (Alexander et al., 2019; Bartman et al., 2016; Benabdallah et al., 2019; Larke et al., 2019 preprint). Regulatory sequences acting at very long range are primarily found in vertebrate species and their discovery was associated with genes displaying complex developmental expression (Grosveld et al., 1987; Lettice et al., 2003; Spitz et al., 2003). In this context, the term ‘regulatory landscape’ was coined to define a large genomic region containing several long-range-acting regulatory sequences that control one or several target genes in a coordinated manner (Spitz et al., 2003).

Since then, many regulatory landscapes have been characterized, in particular in mammals, where they are frequently associated with genes encoding TFs or developmentally important signalling molecules. This may be due to the unusually high functional pleiotropy of such genes, which therefore require batteries of regulatory sequences to control their spatiotemporally complex expression patterns, and to organize and integrate cell fate-specific decisions. As such, regulatory landscapes can contain many transcription units (Ovcharenko et al., 2005; Spitz et al., 2003; Symmons et al., 2014) and genetic studies have suggested that their transcription can be sensitive to structural variations that do not affect the structure of the target genes themselves (Kleinjan and Coutinho, 2009; Spielmann et al., 2018). Accordingly, an increasing number of genetic syndromes affecting embryonic development have been shown to result from the disruption of interactions between regulatory elements and their target promoter, often accompanied by variations in the topological structure of the chromatin (see Lupiáñez et al., 2015).

The recent identification of TADs using experimental approaches based on chromosome conformation capture has further focused attention on how the 3D architecture of the DNA influences gene expression. TADs are relatively small genomic regions (approximately 1 Mb on average) (Bonev et al., 2017), and appear to be produced in part by the zinc-finger protein CTCF and the cohesin complex. The cohesin ring structure envelops a double helix of DNA producing a loop and slides along the DNA until stopped by CTCF proteins present at two locations on the same chromosome (at either end of the TAD), thus forming a loop (Dekker and Mirny, 2016; Nichols and Corces, 2018). The loop regions are stable through development (Rao et al., 2014) and may help insulate regulatory landscapes from interactions with the rest of the genome (Dixon et al., 2016) and at the same time may facilitate very transient enhancer-promoter contacts, providing a dynamic and safe chromatin space where enhancers can act on genes located within a given TAD.

Interestingly, the genomic coordinates of regulatory landscapes often approximate those of TADs, suggesting that the latter may produce a mechanistic boundary to the realm of action of a particular landscape. This relationship is frequently conserved across evolutionary timescales, implicating TADs as a relevant property to gene regulation that can be detrimental to development when disrupted (Harmston et al., 2017; Rao et al., 2014; Symmons et al., 2014). On the other hand, TADs should not be considered as strictly necessary properties of regulatory landscapes: some loci do not rely on a fixed TAD structure, and disruption of TADs may have only a modest effect on gene regulation (Ghavi-Helm et al., 2019; Kragesteen et al., 2018; Rodríguez-Carballo et al., 2019). Thus, the relative importance of TADs for regulatory landscapes and gene transcription remains a matter of open debate.

## A single enhancer dominates regulation

Tissue- and time-specific expression of key developmental genes is generally controlled by the integrated activity of multiple regulatory elements. The concomitant use of several enhancers allows redundancy in function in order to stabilize transcription across space and time, reducing the chance of stochastic haploinsufficiency (Cook et al., 1998; Magee et al., 2003). There are, however, examples for which the loss of a single enhancer phenocopies loss of function of the corresponding gene – at least in the context of a particular tissue. An iconic example of long-range transcriptional regulation involving a single regulatory sequence is the *Shh* gene (Lettice et al., 2003). The limb enhancer ZRS [zone of polarizing activity (ZPA) regulatory sequence] is positioned within the *Lmbr1* gene located 1 Mb upstream from the *Shh* target gene. Homozygous deletion of this enhancer ablates *Shh* transcription in the limb buds, demonstrating that ZRS is necessary for the dosage and tissue specificity of *Shh* transcription in the limb (Sagai et al., 2005). This is supported by the observation that *Shh*-expressing cells in the ZPA display the active-histone modification H3K27ac on the *Shh* gene body and the ZRS enhancer, whereas the entire region between them is devoid of any canonical epigenetic mark indicative of enhancer activity (VanderMeer et al., 2014).

*Shh* and its ZRS enhancer are at opposite ends of the same TAD, yet the 1 Mb distance between them is considerably reduced in 3D space by chromatin folding, a process unaffected by the deletion of the ZRS itself (Amano et al., 2009; Symmons et al., 2016; Williamson et al., 2016). An in-depth genetic analysis of this locus revealed that linear distance and the 3D structure are both relevant for the frequency of interaction between the promoter and the ZRS enhancer, and subsequent *Shh* transcription (Symmons et al., 2016). As such, the loop-extrusion model predicts that CTCF sites surrounding ZRS and *Shh* direct the ZRS towards the *Shh* gene promoter. This was tested by genetic ablation of CTCF sites flanking the ZRS, which reduced the quantity of *Shh* transcripts to approximately half that of wild type. However, deletion of these CTCF-binding sites was not sufficient to breach the threshold for a *Shh* phenotype until the mutation was sensitized with a larger manipulation of the ZRS enhancer sequence (Paliou et al., 2019). These data call into question the influence of CTCF-mediated interactions for this long-range enhancer-promoter interaction. Although CTCF appears to contribute to the interaction, other factors are likely to impact transcription significantly.

The transcription of *Sox9* provides another example of the importance of a single enhancer sequence. During a brief 2-day period in mid-gestation, *Sox9* is upregulated by SRY in male mouse gonads (Hacker et al., 1995). SOX9 then directs the bi-potential gonad towards the male-differentiation pathway, meaning that appropriate activation of *Sox9* is essential to avoid a mismatch between the genetic and the morphological sex (Eggers et al., 2014). The mechanism controlling transcription of *Sox9* appears to be very sensitive to dosage, as a variety of heterozygous regulatory mutations of the locus can cause sex reversal (Bishop et al., 2000; Huang et al., 1999; Kim et al., 2015). The *Sox9* gene is positioned near one end of a 2 Mb large TAD, which contains multiple enhancer elements that can drive transgene expression in the gonads (Franke et al., 2016). However, deletion of each enhancer individually is not sufficient to phenocopy the *Sox9* loss of function, with the notable exception of the *Enh13* sequence (Gonen et al., 2017; Sekido and Lovell-Badge, 2008; Symon and Harley, 2017). This Enh13 enhancer lies 565 kb upstream of *Sox9*, within the ‘XY SR’ region known to cause XY sex reversal in humans when deleted (Gonen et al., 2018). The homozygous deletion of *Enh13* alone reduces *Sox9* transcription to approximately 20% of wild-type XY level, approaching the level found in wild-type XX female gonads, which is obviously not sufficient to determine the male pathway. Sensitivity to the loss of *Enh13* appears to be time dependent: this enhancer acts early to upregulate *Sox9* transcription whereas other local enhancers can subsequently supplement *Enh13* activity. *SOX9* consensus binding sites are found within other enhancers at the *Sox9* locus, suggesting that the *Enh13* enhancer acts first to upregulate *Sox9* transcription above background level, and then the SOX9 protein binds *Enh13* and other enhancers to stabilize *Sox9* transcription in a direct positive-feedback loop (Sekido and Lovell-Badge, 2008). This case illustrates that developing tissues sometimes pass through a bottleneck where precise transcriptional dosage and timing are necessary to trigger the developmental outcome.

Similar to the case of *Sox9*, multiple enhancers stabilize transcription of the hindlimb-determining transcription factor *Pitx1*. Deletion of subsets of these enhancers affect transcription to some extent, yet the homozygous deletion of the Pen enhancer alone reduces transcription to about 55% of control levels, which is sufficient to produce mice with a clubfoot phenotype that mirrors the effect of a heterozygous loss of function of *Pitx1* itself in both mice and humans. Notably, nine CTCF sites are found between *Pitx1* and the Pen enhancer, yet these fail to insulate *Pitx1* from Pen as might be expected from a conventional interpretation of the loop-extrusion model, illustrating the danger of relying exclusively on structural parameters to infer regulatory interactions. Endogenous *Pitx1* is not expressed in forelimbs, but as a transgene, the Pen enhancer can drive transcription in both fore- and hindlimb buds. This is because in forelimbs the Pen enhancer is sequestered by other contacts or titrated out by the closely located *H2afy* (*Macroh2a1*) gene promoter, which prevents interactions with *Pitx1*. In the hindlimbs, the enhancer escapes its local 3D domain to interact with the *Pitx1* promoter, thus allowing transcription and the conferral of hindlimb identity (Kragesteen et al., 2018, 2019).

## Enhancer additivity and cooperativity

In contrast to the examples discussed above, in which a single enhancer dominates regulation, some regulatory landscapes operate using a principle of enhancer additivity, whereby each of several enhancers provides a set percentage of the total transcriptional output produced. In these cases, one would expect a correlation between the number of enhancers deleted and the strength of transcriptional downregulation. However, these effects can be complicated by the varying strength of the individual enhancers that interact with a promoter. It has recently been suggested that weak enhancers may act additively because of their infrequent interaction with the target promoter, but, paradoxically, the combined transcriptional output of multiple strong enhancers may be less than the sum of their individual contributions due to competition and interference between the elements for the promoter (Bothma et al., 2015). In these cases, one cannot predict the contribution of any individual enhancer – and hence the consequences of its deletion – without understanding the global mechanism at work. Despite these complications, single enhancer deletion experiments frequently produce modest reductions in target gene transcription, implicating a conventional additive model in which each enhancer contributes a small and stable regulatory effect (Ahituv et al., 2007; Dickel et al., 2018; Lam et al., 2015; Osterwalder et al., 2018). When no change in transcriptional output is detected, functional redundancy between elements is frequently relied upon for explanation, but there may be a mechanism distinct from mainstream models wherein novel coherent regulatory properties emerge only when the landscape is operating as a whole (discussed below).

The *Ihh* gene – essential for bone growth in the limbs and skull – provides a good example of enhancer additivity. Several upstream enhancer elements have been mapped and transgene analysis showed that the enhancer activities overlap extensively in their expression domains, indicating that each enhancer likely contributes a portion of the enhancer effect in these tissues. Using a set of deletion alleles, it was shown that the quantity of *Ihh* transcription is related to the number of enhancers present, an observation that correlates well with an increase in transcription above wild-type levels when the same enhancers are duplicated (though it should be noted that this increased transcription does not have an overt phenotypic consequence) (Will et al., 2017). Accordingly, the amount of limb shortening observed scales proportionally to the quantity of enhancers present (and their transcriptional output) demonstrating that enhancer addition can produce phenotypes that change with dosage.

Cooperativity has historically been discussed in the context of protein-protein or protein-DNA interactions, but in many cases cooperativity is conferred by the DNA rather than by the proteins themselves and is independent of protein-protein interactions (Jolma et al., 2015; Reiter et al., 2017). When the binding of several TFs to an enhancer is cooperative, the enhancer effect on transcription can increase many times more after the binding of each individual TF (Jolma et al., 2015; Stampfel et al., 2015). Although much of this work has been performed using standardized expression and binding platforms (e.g. GAL/UAS), it may illustrate the dynamics that are observed with endogenous enhancers in the genome.

A variation and notable example of cooperativity observed *in vivo* is the coordinated action of multiple TFs binding at so-called ‘super-enhancers’. This is a type of regulatory landscape that contains extended clusters of closely positioned enhancer elements, each of which can bind multiple TFs that control expression of key cell identity factors (Hnisz et al., 2013; Whyte et al., 2013). Whether or not super-enhancers operate in the same manner as traditional enhancers is debatable (Moorthy et al., 2017; Pott and Lieb, 2014) and, in this context, it is important to distinguish between the cooperativity observed over the large genomic intervals (approximately 15 kb) containing super-enhancers and the cooperativity observed within one traditional ‘enhancer sequence’ (broadly defined as approximately 1 kb in length). In the case of super-enhancers, regulatory sequences may cooperate in a rather non-specific manner, allowing local accumulation of TFs and co-factors, which may help form a functional micro-domain (‘condensate’) spatially isolated from its immediate environment through bio-physical properties (Boija et al., 2018; Hnisz et al., 2017; Sabari et al., 2018).

## Emergent coherence

The colinear expression of Hox genes during limb formation provides a well-studied example of complex spatial and temporal inputs. At both the *HoxD* and *HoxA* loci, appropriate expression requires unique topological arrangements of the gene clusters and their respective long-range enhancers (Andrey et al., 2013; Berlivet et al., 2013; Gentile et al., 2019). At the *HoxD* locus, the TADs flanking the gene cluster match the dimensions of the regulatory regions with the 3′ landscape directing proximal limb expression and the 5′ landscape directing distal limb expression.

Genetic and biochemical assays have identified several enhancer elements within the 5′ regulatory landscape of the *HoxD* locus (Gonzalez et al., 2007; Montavon et al., 2011; Spitz et al., 2003). When these enhancer regions were systematically deleted *in vivo*, Hoxd transcript quantities and distributions were globally affected, but the spatial and quantitative changes varied for each gene and for each deletion (Montavon et al., 2011). This suggests either that the regulatory landscape contains a collection of regulatory elements that differentially affect transcription, or that combinations of enhancers may produce unique pair-wise effects on the transcription of distinct sets of target genes (Fabre et al., 2017). These combinations may vary from cell to cell, or between cellular contexts. Although expression differences between cells may reflect the output of specific enhancer combinations, the global pattern can still be reproducible, perhaps due to a fixed number of potential conformations of the regulatory landscape.

When individual enhancers from these regulatory landscapes were tested as transgenes, they showed expression territories that were generally consistent with their landscape of origin: the transgenes tend to have broader expression domains than expected (Gonzalez et al., 2007; Montavon et al., 2011; Spitz et al., 2003). In light of the examples mentioned above, fully native expression patterns seem to require an intact and coherent 3D structure. TADs may provide a stable framework to this set of 3D structures by limiting the number of possible conformations and also allowing more fluid properties, such as histone modifications and TF content, to define and refine various regulatory potentials.

## Promoter-specific effects

Although regulatory landscapes around developmental genes frequently extend over large gene-poor genomic intervals, they rarely contain a single gene. As a consequence, enhancers can ignore the promoters of some genes – even if they are proximal – while acting specifically on others (Spitz et al., 2003). Several mechanisms may account for this, such as incompatibility between the enhancer and the promoter types (Zabidi et al., 2015). There can be exquisite sensitivity to cellular state within the same tissue, allowing enhancers to switch between two highly similar target genes (Sharpe et al., 1998). Alternatively, one promoter can titrate the enhancer effect away from another promoter in a context-dependent manner (e.g. Cho et al., 2018; Fukaya et al., 2016; Kmita et al., 2002; Lower et al., 2009).

At the *Fgf8* locus, multiple genes are interspersed between *Fgf8* and its enhancers, yet the enhancers do not act on these genes. In this case, there appears to be a structural requirement within the regulatory landscape that defines the promoters with which the *Fgf8* enhancers can interact. Enhancer traps can report the native *Fgf8* expression domains when inserted at various positions within the locus without reporting the ubiquitous expression pattern of flanking genes. However, when the locus is disrupted or portions thereof moved elsewhere, *Fgf8* enhancers act ectopically to drive transcription of the unrelated genes (Marinić et al., 2013). Such an enhancer-promoter re-allocation may result from a disruption to a 3D property that normally sequesters fractions of the locus from the nuclear environment where the enhancers and promoters normally act, allowing these unrelated genes to escape the influence of their surrounding landscape (Marinić et al., 2013). A similar mechanism has been observed at the *Pitx1* locus (see above) (Kragesteen et al., 2018).

There are also cases in which the promoters of two genes positioned in *cis* can act in concert to equilibrate promoter-enhancer contacts and thus modulate the transcriptional output. An intriguing example of this is the *PVT1-MYC* locus. Here, alternative promoters of the long non-coding RNA *PVT1* can capture the activity of enhancers located within its gene body, limiting the effect of these enhancers on the nearby *MYC* promoter. Interfering with one of the *PVT1* promoters by targeted CRISPRi or by deletion of the alternative promoters, as is found in human cancers, prevents the *PVT1* promoter from capturing the enhancer activity, which is therefore redirected to the *MYC* gene causing it to be upregulated and thus promoting growth (Cho et al., 2018).

## Perspectives

The few examples provided above illustrate some of the ways long-range regulations are implemented in regulatory landscapes, in particular at highly pleiotropic loci. There are, however, many instances in which existing models fail to account for the minor, if any, observed effects of enhancer deletion (discussed by Barolo, 2011; Lam et al., 2015). This suggests more than a mere lack of understanding of some details within an otherwise well-accepted explanatory framework. When evaluating experimental observations, several issues should be considered to avoid unnecessary ad hoc conclusions.

First, the results of enhancer-deletion tests should not necessarily be considered together with the outcome of transgenic experiments using the same DNA element, because the two approaches assay different parameters. Transgenic approaches – in which multiple copies of the element are usually present – reveal the potential for a given DNA sequence to elicit a transcriptional response, whereas deletion experiments address the necessity of an element for function. This generally provides insights into the consequences of deconstructing a large and coherent regulatory landscape. In many cases, such landscapes will result from the effects of multiple enhancers' specificities and strengths interacting with structural limitations, and hence the effect of removing one or several such sequences may be hidden or buffered by the remaining landscape. Moreover, a DNA sequence within a landscape may be required for a property that is necessary for the global activity but not directly associated with enhancer specificity, as suggested by the existence of constitutive chromatin interactions even in the absence of transcriptional outcome.

Alternatively, sequences defined as ‘enhancers’ using the criteria outlined above may indeed bind specific proteins following particular logical tendencies (see Long et al., 2016), yet these proteins may not have a direct function influencing the target gene. They may be required to build a general chromatin domain, separated from the nuclear milieu through particular biophysical properties, where genuine enhancers can efficiently interact with their promoters. In such a case, the system would contain an ‘unspecific component’ that is more important than previously anticipated and a genetic dissection approach would need to take this into account (Amândio et al., 2019 preprint). Future technological advances, which will continue to improve sensitivity and specificity in biochemical tests and live optical observation, should help us probe deeper into these issues. Moreover, the increasing number of tools associated with the CRISPR-Cas9 system should help us undertake more complex genomic engineering experiments to assay the activity of particular DNA elements. Thus, although validation by conventional genetic tests will remain the gold standard for evaluating the functional contributions of a regulatory landscape, we will have to consider the results of alternative approaches and move towards a more holistic and perhaps less element-specific understanding of these long-range regulations.
